# Macrophage Migration Inhibitory Factor in Acute Adipose Tissue Inflammation

**DOI:** 10.1371/journal.pone.0137366

**Published:** 2015-09-08

**Authors:** Bong-Sung Kim, Robert Rongisch, Stephan Hager, Gerrit Grieb, Mahtab Nourbakhsh, Hans-Oliver Rennekampff, Richard Bucala, Juergen Bernhagen, Norbert Pallua

**Affiliations:** 1 Department of Plastic and Reconstructive Surgery, Hand Surgery—Burn Center, Medical Faculty, RWTH Aachen University, Aachen, Germany; 2 Department of Medicine, Yale University School of Medicine, New Haven, Connecticut, United States of America; 3 Institute of Biochemistry and Molecular Cell Biology, RWTH Aachen University, Aachen, Germany; Tohoku University, JAPAN

## Abstract

Macrophage migration inhibitory factor (MIF) is a pleiotropic cytokine and has been implicated in inflammatory diseases. However, little is known about the regulation of MIF in adipose tissue and its impact on wound healing. The aim of this study was to investigate MIF expression in inflamed adipose and determine its role in inflammatory cell recruitment and wound healing. Adipose tissue was harvested from subcutaneous adipose tissue layers of 24 healthy subjects and from adipose tissue adjacent to acutely inflamed wounds of 21 patients undergoing wound debridement. MIF protein and mRNA expression were measured by ELISA and RT-PCR. Cell-specific MIF expression was visualized by immunohistochemistry. The functional role of MIF in cell recruitment was investigated by a chemotaxis assay and by flow cytometry of labeled macrophages that were injected into *Mif* ^*–/–*^and wildtype mice. Wound healing was evaluated by an *in vitro* scratch assay on human fibroblast monolayers. MIF protein levels of native adipose tissue and supernatants from acutely inflamed wounds were significantly elevated when compared to healthy controls. MIF mRNA expression was increased in acutely inflamed adipose tissue indicating the activation of MIF gene transcription in response to adipose tissue inflammation. MIF is expressed in mature adipocytes and in infiltrated macrophages. Peripheral blood mononuclear cell migration was significantly increased towards supernatants derived from inflamed adipose tissue. This effect was partially abrogated by MIF-neutralizing antibodies. Moreover, when compared to wildtype mice, *Mif* ^*–/–*^mice showed reduced infiltration of labeled macrophages into LPS-stimulated epididymal fat pads *in vivo*. Finally, MIF antibodies partially neutralized the detrimental effect of MIF on fibroblast wound healing. Our results indicate that increased MIF expression and rapid activation of the MIF gene in fat tissue adjacent to acute wound healing disorders may play a role in cell recruitment to the site of inflammation and wound healing.

## Introduction

Macrophage migration inhibitory factor (MIF) was initially described in the context of delayed-type hypersensitivity [1]. Stored in preformed intracellular pools, MIF release is induced by pro-inflammatory factors such as tumor necrosis factor (TNF)-α, lipopolysaccharide (LPS) or interferon (IFN)-γ [2]. MIF expression in adipose tissue was first reported in the epididymal fat pad of rats and in 3T3-L1 cells, a commonly used murine preadipocyte cell line [3]. Cultured human adipocytes and preadipocytes were found to express a significant amount of MIF which correlated with the body mass index (BMI) of the donor [4]. In 3T3-L1 cells, MIF has an impact on adipogenesis through inhibition of clonal expansion and expression of CCAAT/enhancer binding protein (C/EBP) α and C/EBP δ [5]. Furthermore, MIF mRNA up-regulation is mediated by TNF-α through tyrosine-kinase dependent pathways in 3T3-L1 cells [6].

MIF is a pro-inflammatory protein and a crucial factor in chronic adipose tissue inflammation. Numerous studies have focused on its involvement in chronic adipose tissue inflammation during obesity and insulin resistance while little is known about its role in acute adipose tissue inflammation [7–10].

In this study, we investigated the role of MIF in acute adipose tissue inflammation during wound healing disorders. Wound repair is a complex mechanism of tissue restoration after damage. Not only the epidermis and dermis but also the underlying subcutaneous tissue composed of adipose tissue participates in wound repair [9, 11–13]. Delayed wound healing represents a relevant entity in Plastic Surgery which can lead to life threatening complications and extended soft tissue defects requiring complex reconstruction procedures. Levels of MIF in subcutaneous adipose tissue samples from patients with acute wound healing disorders were measured and compared to MIF levels of healthy adipose tissue. Contrary to its name, MIF exhibits chemokine-like functions and recruits leukocytes into the site of inflammation by binding to its non-cognate receptors CXCR2 and CXCR4 [14]. To better understand the role of MIF in the recruitment of inflammatory cells into adipose tissue and wound healing, a chemotaxis assay, *in vivo* cell tracking, and a scratch assay was performed.

## Methods

### Patients

Whole adipose tissue samples were collected from two groups of patients. The first group consisted of 24 patients (8 male and 16 female, mean age: 50.75 ± 14.75 years) undergoing elective plastic surgery, which included excision of healthy adipose tissue (HAT) from subcutaneous adipose tissue depots (Table 1).

**Table 1:** Details of Healthy Adipose Tissue.

Number	Age	Sex	BMI	Location
1	33	m	29.9	lower extremity
2	54	m	25.9	face
3	56	f	29.4	face
4	24	m	37.9	abdomen
5	65	m	27.2	face
6	71	f	18.7	lower extremity
7	51	m	28.9	abdomen
8	45	f	29.7	abdomen
9	80	f	44.1	lower extremity
10	54	f	35.4	abdomen
11	31	f	35.4	lower extremity
12	39	f	34.9	abdomen
13	54	f	44.6	abdomen
14	48	m	27.2	upper extremity
15	54	f	25.4	upper extremity
16	74	m	40.12	lower extremity
17	51	f	33.80	upper extremity
18	74	f	30.35	face
19	51	m	32.27	upper extremity
20	38	f	36.00	upper extremity
21	29	f	27.34	lower extremity
22	43	f	28.34	upper extremity
23	56	f	24.80	abdomen
24	43	f	34.05	abdomen

Age, Sex, BMI, and Harvesting Location of Healthy Adipose Tissue.

The second group included 21 patients (9 male and 12 female, mean age: 52.62 ± 16.14 years) with acute wound healing disorders (Table 2).

**Table 2:** Details of acutely inflamed adipose.

Number	Age	Sex	BMI	Location	Specification
1	53	f	25.4	upper extremity	wound healing disorder after external trauma
2	25	f	23.4	face	postoperative wound healing disorder
3	56	m	17.9	lower extremity	Iatrogenic superficial thrombophlebitis
4	68	m	27.8	upper extremity	Iatrogenic superficial thrombophlebitis
5	42	m	19.6	upper extrimity	wound after external trauma
6	69	m	27.8	lower extremity	Iatrogenic superficial thrombophlebitis
7	70	m	34	upper extremity	postoperative wound healing disorder
8	38	f	34.72	upper extremity	wound healing disorder after external trauma
9	39	f	34.9	abdomen	postoperative wound healing disorder
10	74	m	38	lower extremity	postoperative wound healing disorder
11	40	f	89	abdomen	postoperative wound healing disorder
12	40	m	32.1	abdomen	postoperative wound healing disorder
13	52	f	19.4	upper extremity	wound healing disorder after external trauma
14	55	m	27.2	lower extremity	Iatrogenic superficial thrombophlebitis
15	40	m	32.1	upper extremity	postoperative wound healing disorder
16	57	f	29.4	upper extremity	postoperative wound healing disorder
17	80	f	33.3	abdomen	postoperative wound healing disorder
18	74	m	40.12	lower extremity	wound healing disorder after external trauma
19	66	f	58.88	lower extremity	wound healing disorder after external trauma
20	38	f	34.89	lower extremity	wound healing disorder after external trauma
21	29	f	27.34	upper extremity	wound healing disorder after external trauma

Age, Sex, BMI, Harvesting Location, and Specification of acutely inflamed adipose.

These were defined as wounds either caused by external trauma, iatrogenic manipulation or after surgical intervention that showed the classic signs of local inflammation (*tumor*, *calor*, *rubor*, *dolor* and *functio laesa*), negative bacterial swab samples at the time of tissue harvest, did not exceed a time period of four weeks after trauma or intervention, and needed surgical debridement. Small blocks of acutely inflamed adipose tissue (IAT) were excised within a distance of 1 cm to the wound margin. Necrotic fat tissue was discarded. Samples were immediately brought to the laboratory where adipose tissue was minced with scissors. After meticulous removal of blood vessels and connective tissue, the samples were washed and stored for later analysis. Supernatants were collected from adipose tissue cultivated in medium (DMEM/Ham’s F-12 supplemented with 0.5% fetal calf serum (FCS), 100 U/mL penicillin, 100 μg/mL streptomycin and 1 ng/ml bFGF) for 24 hours. All surgeries were performed in the Department of Plastic Surgery, Hand Surgery–Burn Center of the RWTH University Hospital Aachen, Germany. The use of human samples was approved by the ethics committee of the RWTH Aachen University (EK 163/07). All patients provided written consent. All experiments were performed in compliance with the Declaration of Helsinki Principles.

### Detection of total protein contents

Total protein content was measured by the DC Protein Assay (BioRad Laboratories GmBH, Munich, Germany) according to the manufacturer’s instructions. The colorimetric reaction was detected at 740 nm using the FLUOstar OPTIMA microplate reader (Fluostar Optima; BMG Labtech, Offenburg, Germany). MIF levels of whole adipose tissue samples assessed by ELISA were normalized to the total protein contents of each sample.

### Sandwich-enzyme-linked immunosorbent assay (ELISA)

ELISA of homogenized whole adipose tissue and supernatants was performed with MAB289 capture (R&D Systems, Abingdon, United Kingdom) and BAF289 detection antibodies (R&D Systems) as a sandwich-ELISA. The tissue used for our studies was not further manipulated, digested with collagenase, or cultured and therefore represents the native state.

Each well was coated overnight with primary antibody solution at a final concentration of 2 μg/ml. Plates were blocked with PBS containing 1% BSA and 5% sucrose for one hour. After incubating samples for another two hours, secondary antibody was added at a final concentration of 200 ng/ml. Streptavidin-POD enzyme conjugate (R&D Systems, Abingdon, United Kingdom, 1 μl/10 ml TBS) was added and left for 20 minutes. Color reaction was determined by the Substrate Reagent Pack DY999 by R&D Systems (R&D Systems, Abingdon, United Kingdom). The reaction was terminated after 20 minutes by adding 0,5 M H_2_SO_4_ in ddH_2_O and plates were read at 450 nm (FLUOstar OPTIMA microplate reader). Bacterially expressed MIF was used as a standard. All assays were carried out in duplicates.

### Real time-polymerase chain reaction (RT-PCR)

mRNA from whole adipose tissue for RT-PCR was prepared by the QIAzol Lysis Reagent (Qiagen NV, Venlo, Netherlands) and the RNeasy Mini Kit (Qiagen NV, Venlo, Netherlands) following the manufacturer’s instructions.

cDNA synthesis was performed using the First Strand cDNA Synthesis Kit (Thermo Fisher Scientific Inc, Waltham, USA) following the manufacturer’s instructions.

The resulting cDNA was subject to quantitative RT-PCR using the 2x SensiMix SYBR No-ROX Kit (Peqlab Biotechnology, Erlangen, Germany), cDNA template, human MIF-Primer (MIF-Forward: CCGGACAGGGTCTACATCAACTATTAC, MIF-Reverse: TAGGCGAAGGTGGAGTTGTTCC) and glyceraldehyde 3-phosphate dehydrogenase (GAPDH)-Primer (GAPDH-Forward: GCCTCAAGATCATCAGC, GAPDH-Reverse: ACCACTGACACGTTGGC) as an internal control with an annealing temperature of 60°C (Primer: Eurofins MWG Operon, Ebersberg, Germany and Sigma-Aldrich Chemie GmbH, Munich, Germany). Measurements were conducted with the RotorGene 6000 (Qiagen NV, Venlo, Netherlands).

### Immunohistochemistry

Adipose tissue was fixed in 10% formaldehyde, dehydrated and embedded in paraffin. Next, samples were deparaffinized and rehydrated. To regain immune reactivity, heat induced epitope retrival (IZKF Biomat.-Histofacility, UK Aachen) was performed. To block non-specific background staining, DAKO Protein Block (DAKO Deutschland GmbH, Hamburg, Germany) was used.

For fluorescent staining, the primary mouse anti-human-MIF antibody MAB 289 (R&D Systems, Abingdon, United Kingdom) as well as Alexa Fluor 488 dye (Life Technologies GmbH, Darmstadt, Germany) was added. Nuclear staining was performed with 4',6-diamidino-2-phenylindole (DAPI).

### Isolation and cultivation of peripheral blood mononuclear cells (PBMC)

PBMCs were isolated from buffy coats obtained from the Transfusion Medicine/Blood Donation Service of the RWTH-Aachen University Hospital by the Ficoll-hypaque density-gradient centrifugation method. Isolated cells were cultured in RPMI 1640 medium containing 100 U/mL penicillin, 100 μg/mL streptomycin, and supplemented with 10% FCS. After three days of culture, adherent PBMCs were used for the chemotaxis experiments.

### Chemotaxis assay

PBMC migration was evaluated in 96-well Transwell-plates (pore size: 5 μm, Corning HTS Transwell-96, Corning, USA). Medium containing 10% FCS served as a positive control (PC), medium containing 0.5% FCS served as a negative control (NC). Supernatants of HAT or IAT were collected in medium containing 0.5% FCS. MIF and monocyte chemoattractant protein (MCP)-1-dependent effects were tested by use of anti-MIF antibodies (NIH/III.D9) and anti-MCP-1 antibody (Biolegend, San Diego, United States), respectively. The bottom chambers of the 96 well plate were filled with PC, NC, and samples plus antibodies. Next, 5x10^4^ PBMCs were added into the upper chamber and the whole plate was incubated for 2.5 hours at 37°C and 5% CO_2_. For evaluation, serial images of migrated PBMCs in the bottom chamber were obtained with an inverted phase contrast microscope (Leica DMI4000 B) followed by cell counting with the ImageJ software (National Institute of Health, Bethesda, USA).

### *In vivo* model

#### Mice

Ten to 12-week-old male C57BL/6 wildtype (WT) and *Mif* ^*–/–*^mice were used in this study. WT mice were purchased from Charles River Laboratory (Wilmington, United States). The generation of *MIF* ^*–/–*^mice was reported earlier [15]. Animals were housed in plastic cages fed with standard chow and water *ad libitum*. All animal experiments were approved by Yale University's Institutional Animal Care and Use Committee (2015–10756).

#### Macrophage harvest and labeling

Macrophages were collected from WT mice after intraperitoneal injection of 5% thioglycollate by peritoneal lavage. Harvested macrophages were then washed followed by erythrocyte lysis by ACK lysing buffer (BioWhittaker, Walkersville, United States) and finally labeled with fluorescein isothiocyanate (FITC) (Cell Tracker Green CMFDA Dye, Life Technologies, Carlsbad, United States).

#### *In vivo* migration after LPS injection

5 mg/kg LPS (Sigma Aldrich, St. Louis, United States) was injected into the epididymal fat pads of WT and *Mif* ^*–/–*^mice via a small incision followed by retro-orbital injection of 2 x 10^6^ FITC-labeled macrophages. After 48 h, mice were sacrificed and epididymal fat tissue harvested.

#### Flow cytometry

The content of FITC-positive macrophages in the harvested adipose tissue was measured by flow cytometry as described previously [16]. In short, adipose tissue was minced and stromal vascular fraction (SVF) cells yielded by digestion with Collagenase Type II (Sigma Aldrich, St. Louis, United States). Following antibodies were used for surface staining: CD11b-AlexaFluor700 (eBioscience, San Diego, United States), F4/80-eFluor450 (eBioscience, San Diego, United States), CD45-APC (Biolegend, San Diego, United States). Analyses were performed on a LSR II cytometer (BD Bioscience, San Jose, United States).

#### *In vitro* wound healing assay

Human dermal fibroblasts were acquired from the Yale Human Cell Resource Center and cultured in Dulbecco's Modified Eagle Medium (DMEM) containing 100 U/mL penicillin, 100 μg/mL streptomycin, and supplemented with 10% FCS [17]. Wound healing of monolayers of human dermal fibroblasts was evaluated by a standardized scratch assay following a previous protocol with medium containing 10% FCS serving as a PC and medium containing 0% FCS serving as a NC [18]. Fibroblasts were seeded to 80% confluence in 24-well cell culture plates. Cell monolayers were scratched with a p200 pipette tip. After washing the plates with growth medium, fibroblasts were incubated with sample supernatants for 16 hours, MIF dependency was evaluated by anti-MIF antibodies (NIH/III.D9) and the respective isotype control. Experiments were further performed in the presence and absence of 10μg/ml Mitomycin C (Sigma Aldrich, St. Louis, United States) to discern between proliferation and migration-related wound healing. Microscopic photographs were taken at 0 hours and 16 hours. Reference points were marked on the bottom of the plate close to the scratch by a scalpel for exact adjustment during image acquisition [19]. The relative migration (in the presence of Mitomycin C) and migration plus proliferation distance (in the absence of Mitomycin C) was deduced from the comparison of the 0 hour and 16 hours pictures by the Software Photoshop CS5 (Adobe Systems, CA, USA) and presented in arbitrary units.

### Statistical analysis

Flow cytometry data were analyzed by the software FlowJo (Ashland, United States). For all statistical analysis and diagrams, the software GraphPad Prism (GraphPad Software, Inc., La Jolla, USA) was used. All data were expressed as mean ± SEM. Statistical significance was calculated by one-way ANOVA in case of three or more variables and t-test in case of two variables with a p value of < 0.05 considered as significant. Associations between MIF protein levels from native adipose tissue and the donor’s BMI was described by Spearman’s rank correlation with calculation of linear regression and 95% confidence interval.

## Results

### MIF protein levels are increased in native subcutaneous adipose tissue from IAT

**Fig 1:**
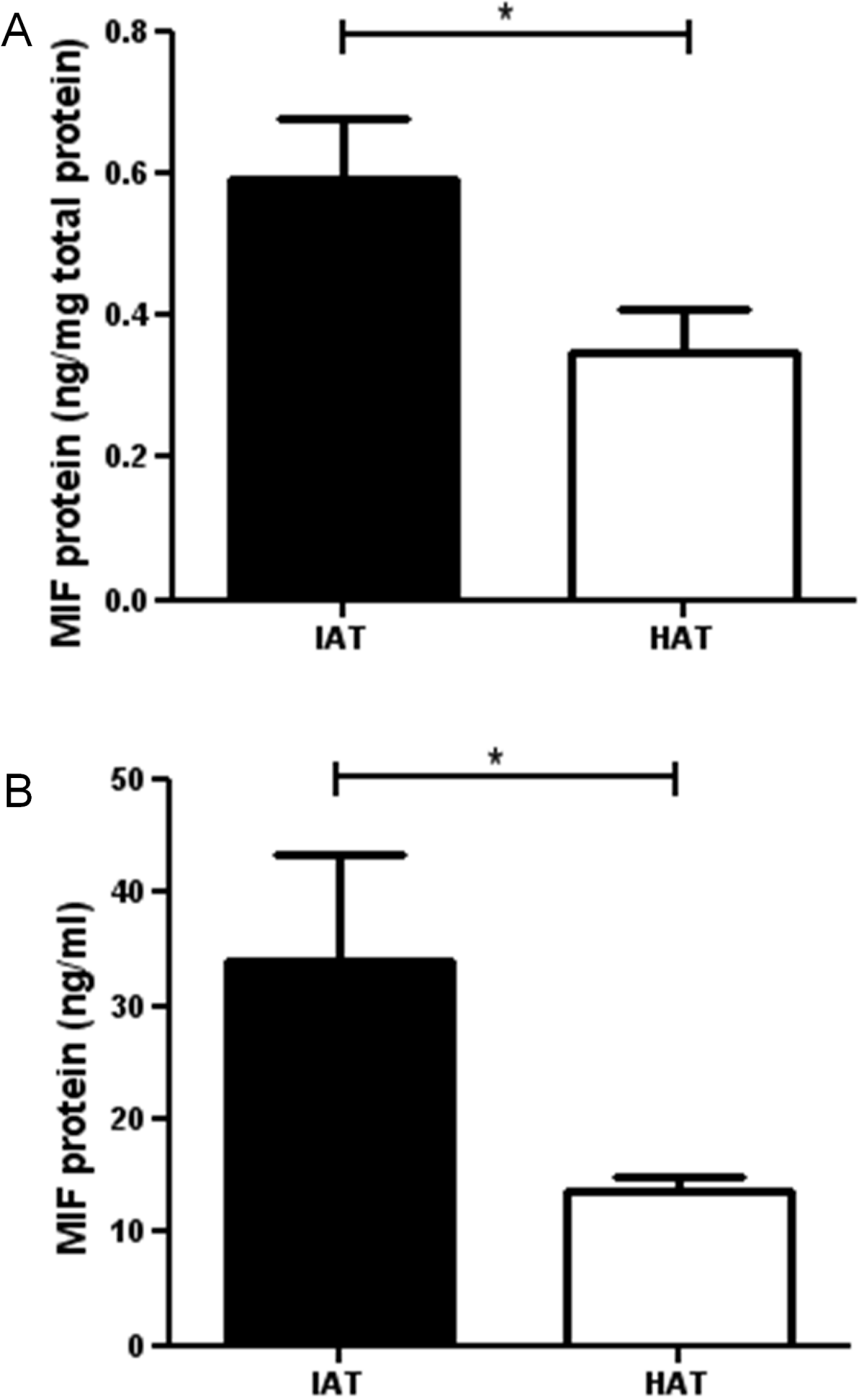
Levels of MIF in native HAT and IAT. Adipose tissue from healthy donor sites and acutely inflamed donor sites were homogenized. MIF levels were measured by ELISA. **A** MIF levels of native HAT and IAT **B** MIF levels of supernatants derived from HAT and IAT. Data are presented in mean MIF concentration ± SEM. Statistically significant differences are indicated by asterisks (*, P<0.05).

We first measured the level of MIF protein in subcutaneous adipose tissue from healthy donors. Subcutaneous adipose tissue samples of healthy donor sites contain significant amounts of MIF (Fig 1A). Next, subcutaneous tissue samples from acutely inflamed donor sites were investigated for their respective MIF content. Mean levels of MIF protein in IAT were almost two-fold higher compared to HAT (Fig 1A). The difference was statistically significant. Similarly, the supernatants of acutely IAT secreted significantly higher amounts of MIF than supernatants from HAT (Fig 1B).

### MIF mRNA expression is increased in native subcutaneous adipose tissue from IAT

**Fig 2:**
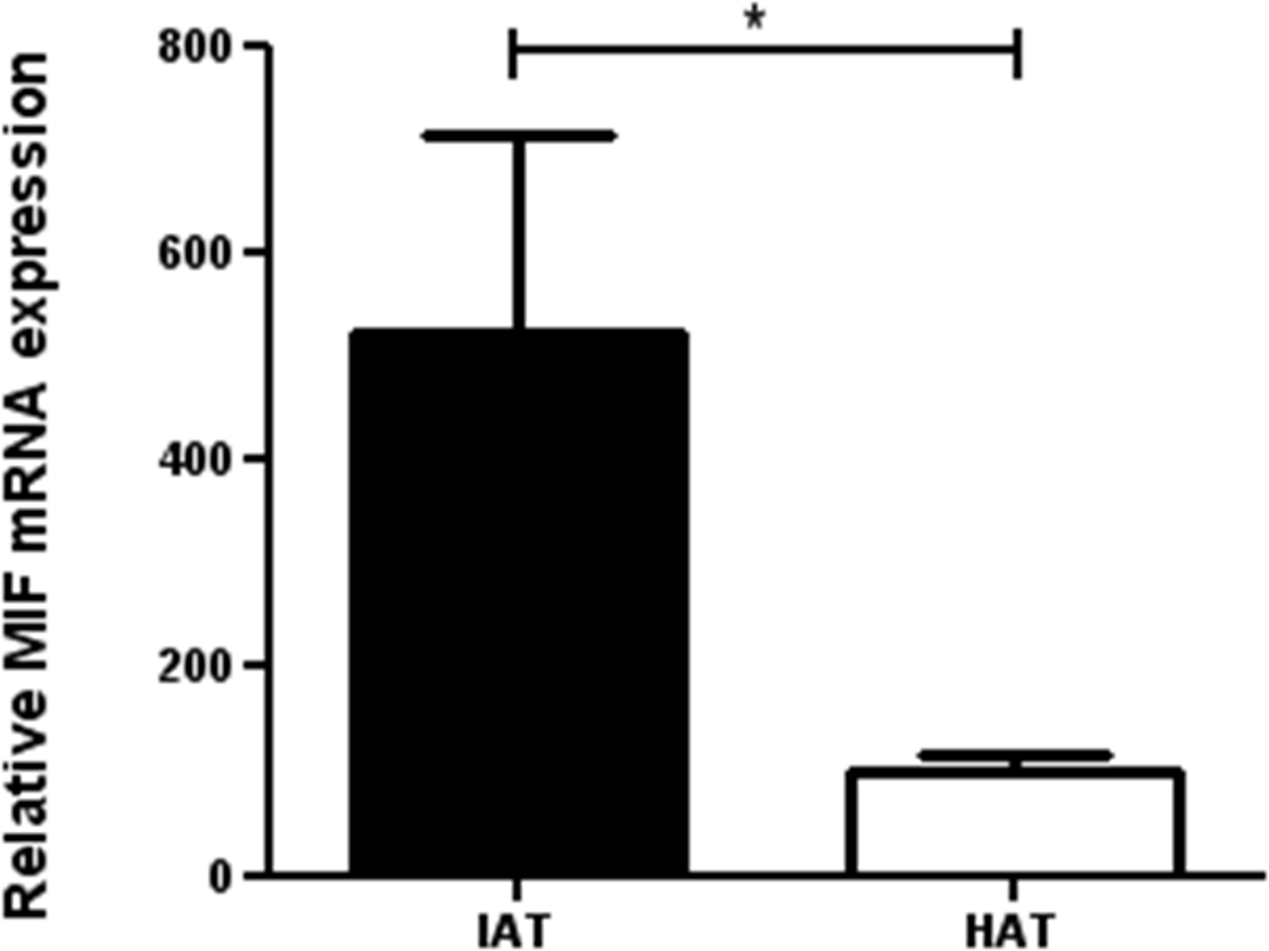
Relative MIF-mRNA expression in HAT and IAT. HAT and IAT were homogenized. MIF-mRNA levels were measured by RT-PCR. The mean value for MIF gene expression for HAT was standardized to a relative mean value of 100%. Data are presented in mean % of HAT ± SEM. Statistically significant differences are indicated by asterisks (*, P<0.05).

The expression of MIF mRNA of native HAT and IAT was performed by RT-PCR (Fig 2). The housekeeping gene GAPDH served as an internal control. To relate the data of the control group to the data of the experimental group in an overall context, the mean value for MIF gene expression for the control group was standardized to a relative mean value of 100%. The mean value of the experimental inflammatory group was multiplied by the same factor.

RT-PCR revealed expression of MIF-mRNA in adipose tissue (Fig 2). Importantly, the mean value for MIF gene expression in IAT was more than five times higher when compared to HAT. The difference was statistically significant.

### MIF is localized in the cytoplasm of native adipocytes and adipose tissue macrophages (ATM)

**Fig 3:**
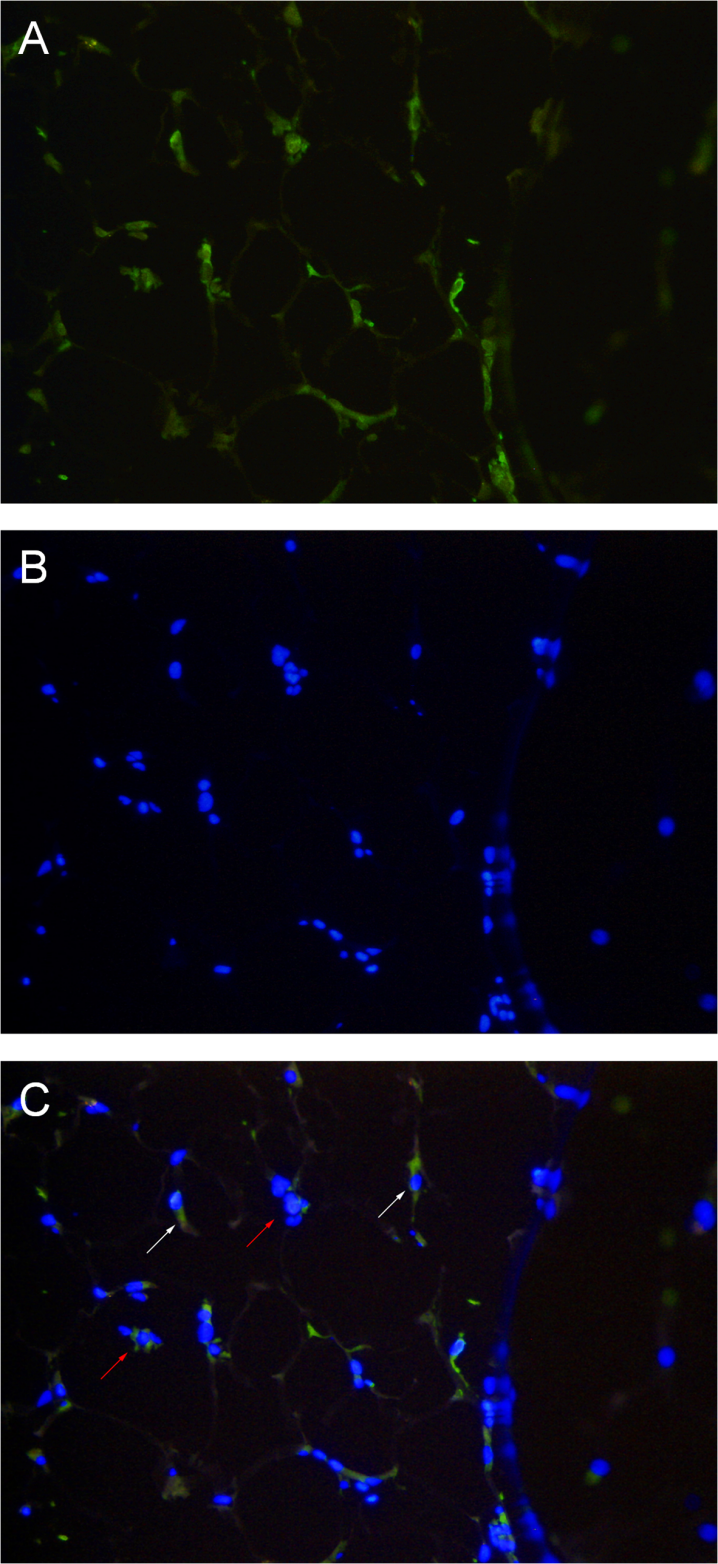
MIF expression in adipocytes and ATM of HAT and IAT. MIF expression was evaluated by immunohistochemistry on formalin fixed human fat tissue (200x magnification). MIF is detectable in single adipocytes (red arrows) and in ATMs infiltrated as crown-like structures (white arrows). **A** DAPI staining **B** MIF-Alexa 488 staining **C** Overlay of DAPI and MIF-Alexa 488.

MIF expression was evaluated by immunohistochemistry on formalin fixed human fat tissue. As demonstrated in Fig 3, MIF is detectable in the cytoplasm of adipocytes. Furthermore, MIF was localized in the cytoplasm of ATMs which infiltrated the adipose tissue and formed crown-like structures. It is important to note, that MIF protein was detectable only in a subset of adipocytes and ATMs. Thus, it is conceivable that MIF protein is differentially expressed throughout human fat tissue.

MIF staining of the lipid vacuoles was not possible due to the fixation process which led to a complete evacuation of the vacuoles. The comparison of HAT and IAT did not lead to visible differences in MIF expression of adipocytes or macrophages but showed generally higher ATM infiltration.

### MIF protein levels of HAT but not IAT correlates with the donor’s BMI

**Fig 4:**
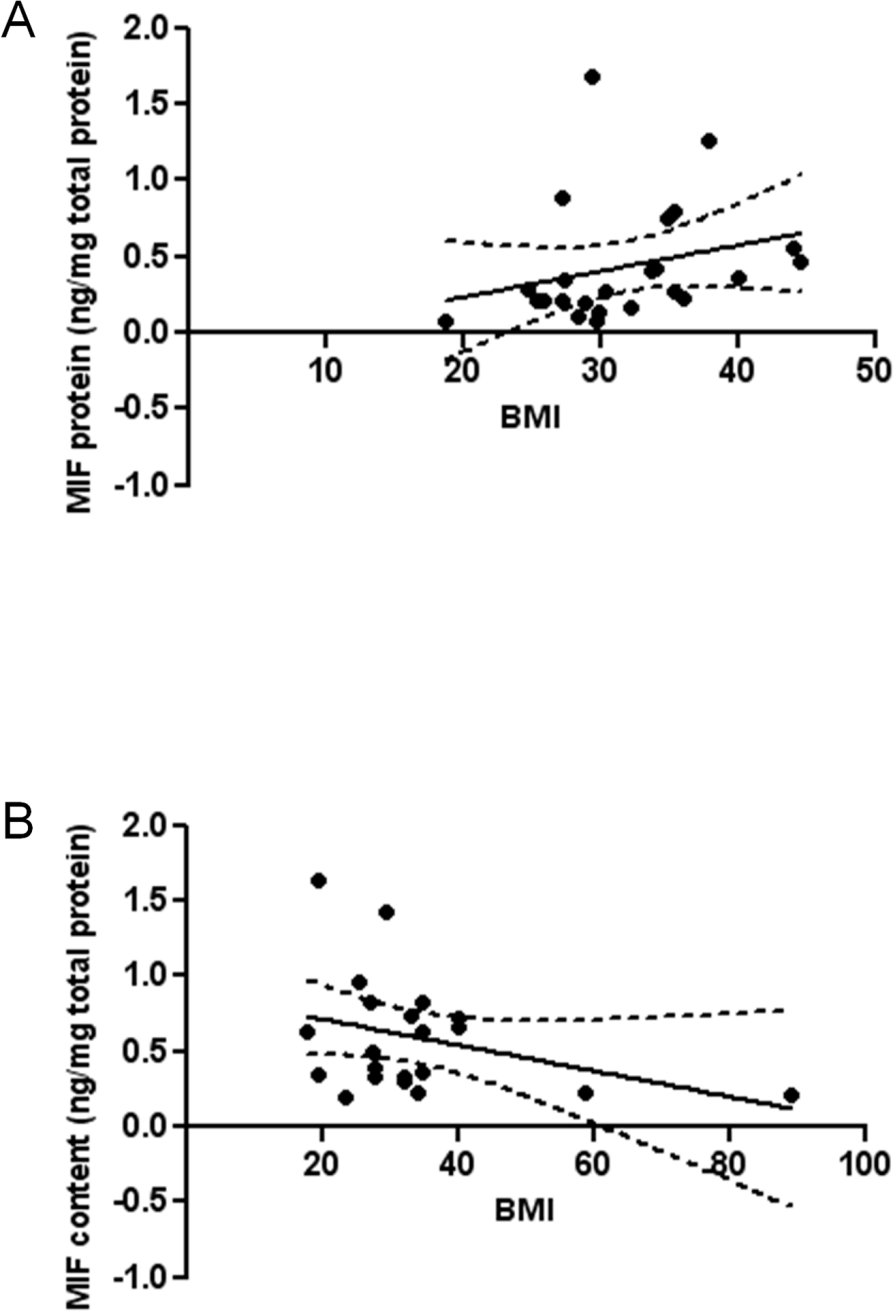
Correlation of MIF protein with donor’s BMI. MIF protein levels from native adipose tissue were correlated (Spearman’s rank order correlation) with the BMI of the donor. The linear regression curve is depicted by a solid line, the 95% confidence interval by a dotted line. **A** MIF protein levels and BMI from donors of HAT show a significant positive correlation (p<0.05, Spearman r = 0.39). **B** MIF protein levels and BMI from donors of IAT show no significant correlation (p = 0.32, Spearman r = -0.23).

Next, we correlated MIF protein levels from native adipose tissue with the BMI of the donor by Spearman’s rank order correlation and calculated the regression curve with the 95% confidence interval. MIF protein levels of HAT positively correlate with the donor’s BMI (Fig 4A). The correlation was statistically significant (p<0.05, Spearman r = 0.39). No significant correlation was seen in MIF protein levels of IAT and their donor’s BMI (p = 0.32, Spearman r = -0.23) (Fig 4B).

### PBMC migration is increased towards supernatants derived from IAT

**Fig 5:**
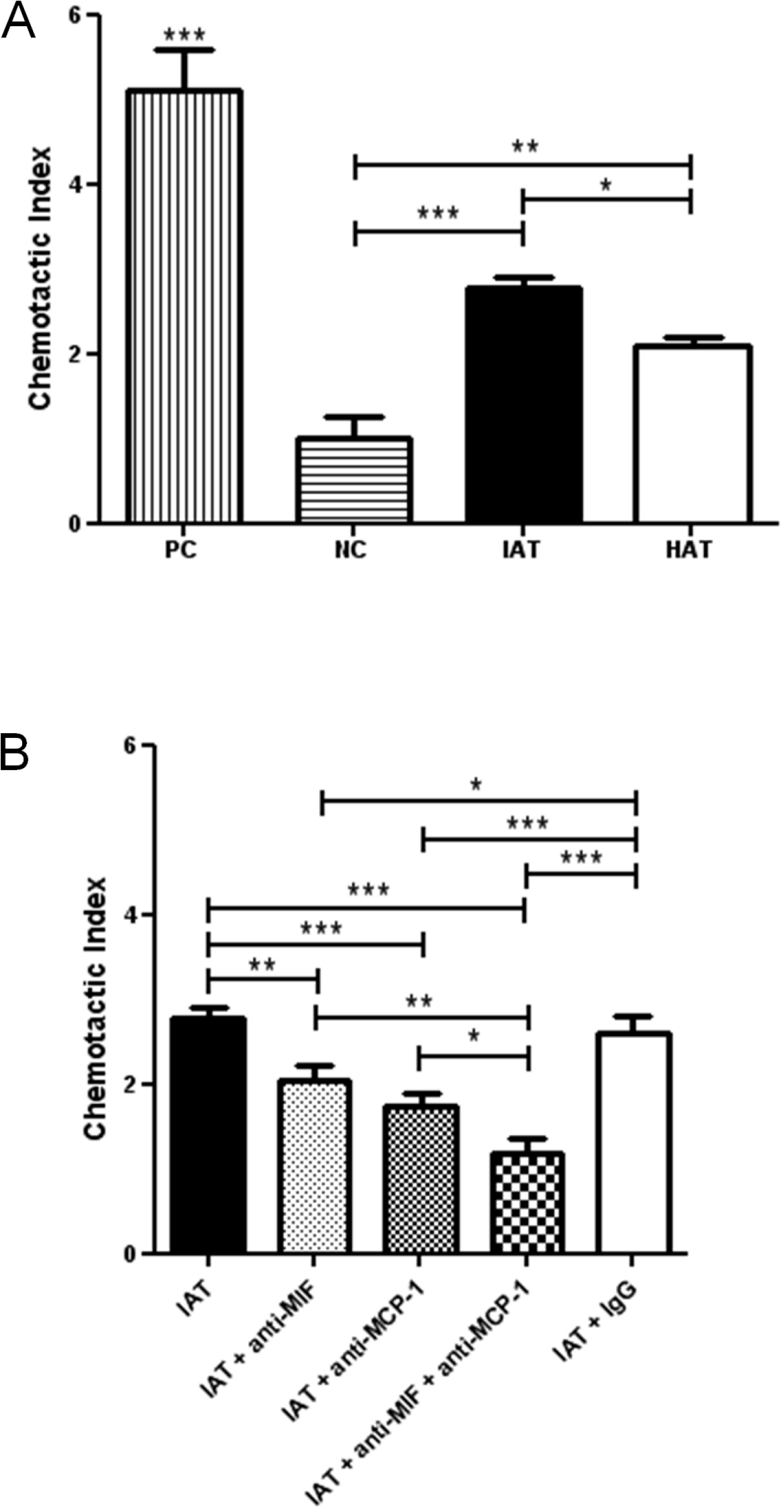
Chemotaxis of PBMC towards supernatants from HAT and IAT. PBMC migration was evaluated in 96 well Transwell-plates (pore size: 5 μm). Medium containing 10% FCS served as a positive control (PC), medium containing 0.5% FCS served as a negative control (NC). Supernatants of HAT and IAT were collected in medium containing 0.5% FCS. MIF and MCP-1-dependent effects were shown by use of monoclonal anti-MIF and anti-MCP-1 antibody and an IgG isotype control. The chemotactic index represents the ability of the sample to induce cell migration. Each sample was normalized to the negative control. Data are presented in mean chemotactic index ± SEM. Statistically significant differences are indicated by asterisks (*, p<0.05; **, p<0.01; ***, p<0.001). **A** PBMC migration towards supernatants of HAT and IAT compared to PC and NC. **B** PBMC migration toward IAT partially neutralized by MIF and MCP-1 antibodies.

In order to underscore the functional consequences of our observations, we conducted an *in vitro* chemotaxis assay (Fig 5). Supernatants of subcutaneous tissue from HAT and IAT were used to investigate the migration capacity of PBMCs. PBMC migration was higher in response to supernatants from IAT when compared to supernatants derived from HAT. These results indicate that soluble factors from the supernatants of HAT and IAT both secrete soluble factors that stimulate PBMC migration with the inflamed tissue having a higher chemotactic potency.

### PBMC migration is partially abrogated by MIF antibodies

We used anti-MIF and anti-MCP-1 antibodies to examine whether MIF and/or MCP-1 contributed to the pro-migratory effects of the IAT and HAT supernatants. The addition of MIF and MCP-1 antibodies significantly disrupted PBMC migration (Fig 5). Anti-MCP1 and anti-MIF both showed partial inhibition, with the effect being in the same order of magnitude. When both antibodies were given simultaneously an additive effect was observed.

### *In vivo* macrophage migration is lower in Mif^–/–^mice

**Fig 6:**
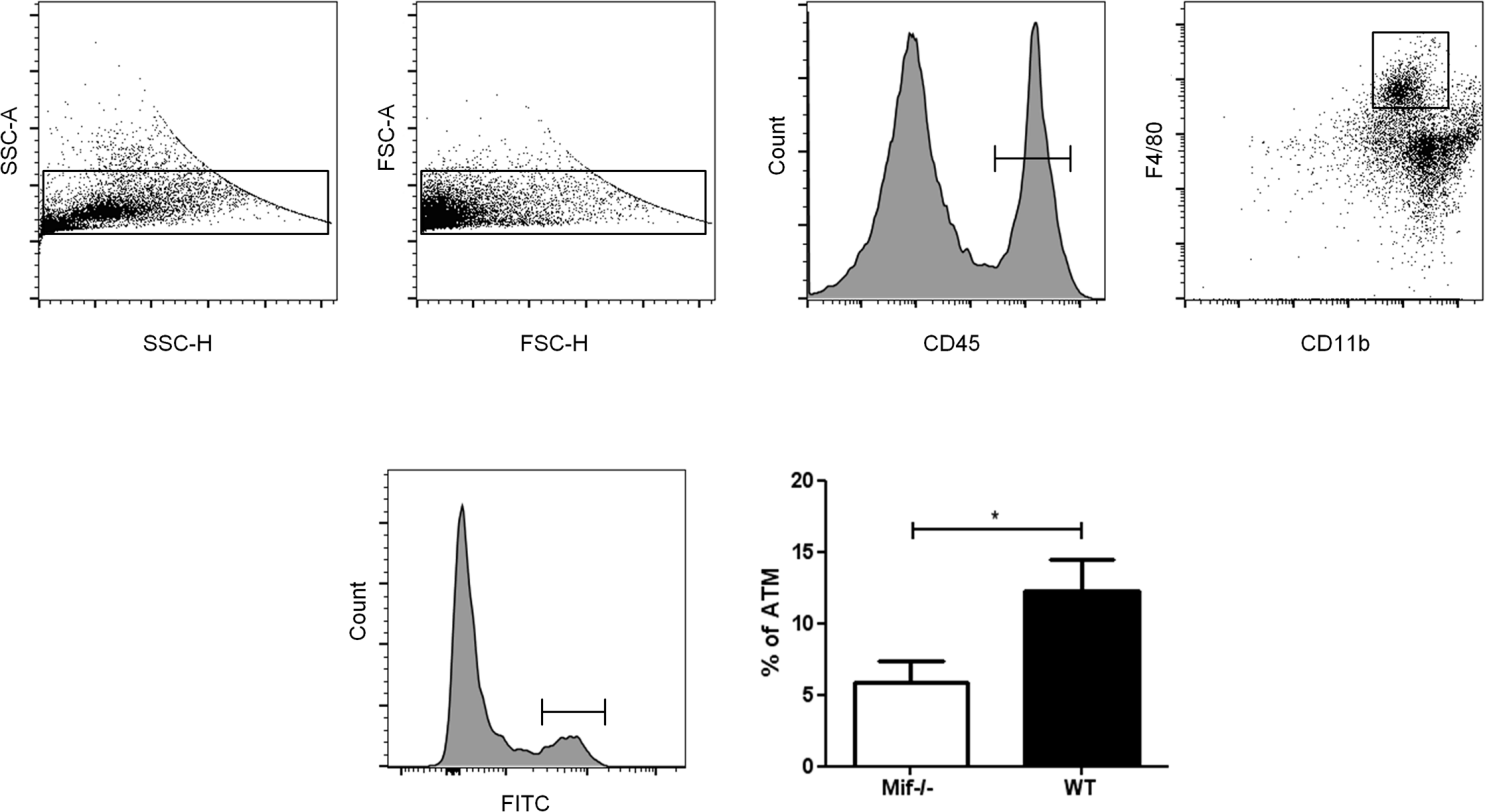
Flow cytometry of labeled ATM from epididymal fat pads from WT and *Mif* ^*-/-*^ mice. FITC-labeled macrophages were injected retro-orbitally into WT and *Mif* ^*-/-*^ mice after inducing a local inflammation in the epididiymal fat pads by LPS. After 48 hours, mice were sacrificed and FITC-positive macrophages were measured by flow cytometry. Doublets were excluded by sideward scatter (SSC) and forward scatter (FSC), macrophages were defined as CD45, CD11b, and F4/80 positive cells. The results show a significant lower number of FITC-positive macrophages in *Mif* ^*-/-*^ mice when compared to WT mice. Data is presented as % of FITC-positive ATMs ± SEM. Statistically significant differences are indicated by asterisks (*, p<0.05).

In order to further elucidate the functional role of MIF in inflammatory adipose tissue, we performed an *in vivo* cell tracking experiment. This method was previously reported for the measurement of ATM accumulation in obese mice [20]. Labeled macrophages were injected into WT and *Mif* ^*–/–*^mice after inducing a local inflammatory response in the epididiymal fat pads by LPS. FITC-positive macrophages recovered from the inflamed fat pads were measured by flow cytometry. Macrophages were defined as CD45-, CD11b-, and F4/80-positive cells. Results show a significantly lower number of labeled macrophages in *Mif* ^*–/–*^mice when compared to WT mice (Fig 6). Macrophage infiltration of visceral fat was markedly lower in visceral adipose tissue depots of *Mif* ^*–/–*^and WT mice.

### Adipose tissue-derived MIF partially inhibits fibroblast migration in an *in vitro* scratch assay

**Fig 7:**
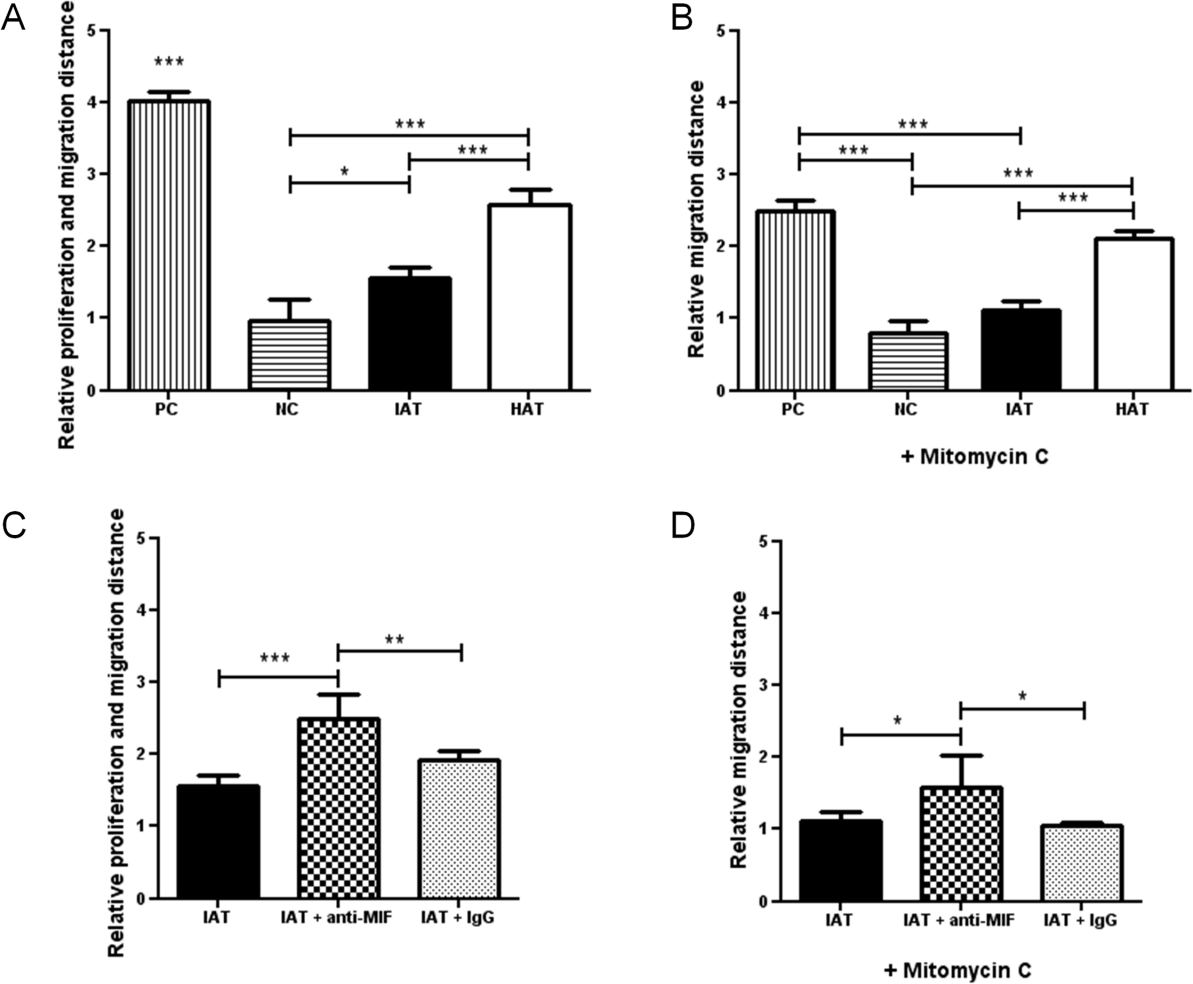
*In vitro* scratch assay of dermal fibroblasts and supernatants from IAT and HAT. Wound healing of human dermal fibroblast monolayers was evaluated by an *in vitro* scratch assay. Fibroblasts were incubated with supernatants of samples from IAT and HAT, MIF-dependency was evaluated by anti-MIF antibodies. Medium containing 10% FCS served as a PC and medium containing 0% FCS served as a NC. Experiments were performed in the presence (A, B) and absence (C, D) of the proliferation inhibitor Mitomycin C. **A** Supernatants from IAT show a decreased induction of fibroblast proliferation and migration (no Mitomycin C). **B** Fibroblast proliferation and migration is increased by addition of MIF-antibodies (no Mitomycin C). **C** Supernatants from IAT show a decreased induction of fibroblast migration (10 μg/ml Mitomycin C). **D** Fibroblast migration is increased by addition of MIF-antibodies (10 μg/ml Mitomycin C). All measurements were normalized to the negative control of Fig 7A which was set as 1. Data are presented in relative migration distance of fibroblasts ± SEM. Statistically significant differences are indicated by asterisks (*, p<0.05; **, p<0.01; ***, p<0.001).

An *in vitro* scratch assay was used to evaluate the role of adipose tissue-derived MIF on the healing response of fibroblasts. Human dermal fibroblast monolayers were scratched and incubated with supernatants of IAT and HAT. Each experiment was conducted in the presence and absence of the potent proliferation inhibitor Mitomycin C in order to discern wound healing by proliferation and migration *versus* migration only. In the absence of Mitomycin C, supernatants from HAT elicited a significantly higher proliferation and migration of scratched fibroblast monolayers than IAT (Fig 7). The addition of an anti-MIF antibody significantly led to a recovery of the proliferation and migration of fibroblasts incubated with IAT supernatants. When proliferation of fibroblasts was eliminated by addition of Mitomycin C, overall wound healing was decreased but the effect of anti-MIF remained significant. The results indicate that MIF has an impact on the migration of fibroblasts.

## Discussion

Wound healing is a sequence of complex processes and depends on a myriad of factors. Fat tissue is a critical organ in body homeostasis and the increasing number of obese patients with impaired wound healing has generated significant interest in the function of adipose tissue during wound repair [13, 21]. Subcutaneous adipose tissue as the tissue layer in immediate proximity to the wound or even exposed in deep tissue defects plays a role in wound healing through soluble factors. In inflamed adipose tissue, impaired vascularity, oxygen tension, infiltration by inflammatory cells, cellular as well as molecular alterations jointly have a significant impact on wound healing. This observation is evidenced by the delayed and poor wound healing that can occur in obese patients [13, 22]. The regenerative effect of adipose tissue on keratinocyte migration, proliferation, and differentiation or on fibroblasts was documented early [9, 11, 12, 23]. In particular, adipose precursor cells, so called adipose-derived stem cells (ASC), appear to have a high regenerative capacity in wounds [24, 25].

In this study, we measured MIF protein levels by ELISA and *MIF* expression by RT-PCR in human subcutaneous adipose tissue. In contrast to Fain *et al*. [7] and also Skurk *et al*. [4], who both measured the release of MIF by cultured adipocytes, adipose tissue matrix, stromal vascular fraction, or ASCs, our approach focused primarily on the native status of adipose tissue. Additionally, we localized MIF in the cytoplasm of native, mature human adipocytes and ATMs. MIF was hitherto only stained in cultured human adipocytes, preadipocytes or in the epididymal fat pad of rats [3, 4].

We further studied the role of MIF in adipose tissue inflammation and mobilization of inflammatory cells. To date, studies of the role of adipose tissue in inflammation have mainly concentrated on its function in chronic inflammation during obesity and diabetes mellitus. Koska *et al*. observed a positive association of MIF with adipocyte diameter as a measuring unit for obesity accompanied by a negative correlation to hepatic and peripheral insulin action in primary human adipocytes and cultured preadipocytes [26]. Interestingly, no comparable correlations were observed for MCP-1, MCP-2, macrophage inflammatory protein (MIP)-1α, MIP1-β, MIP-2, TNF-α, interleukin (IL)-6 or IL-8. Elevated serum MIF levels were described in obese female patients, suggesting a role for MIF as a potential contributor for the metabolic syndrome [27]. MIF is the only factor amongst interleukin 1 receptor antagonist (IL-1Ra), cathepsin S, nerve growth factor (NGF) and IL-18 showing increased release in morbidly obese patients as described by Fain *et al*. We show that the MIF content of healthy subcutaneous fat tissue correlates with the BMI from the donors underpinning the role of MIF in obesity. No significant correlation, however, is seen between MIF protein levels of IAT and the donor’s BMI. The acute up-regulation of MIF in adipose tissue in inflamed wounds appears to mask the aforementioned correlation.

We highlighted the role of MIF in acute subcutaneous adipose tissue inflammation in the context of acute wound healing disorders. We demonstrate that subcutaneous adipose tissue harvested from acutely inflamed wounds show a significantly higher MIF content and expression than subcutaneous adipose tissue harvested from healthy donor sites. Increased local expression of MIF was observed in tissue samples from venous ulcers and Emmerson *et al*. further showed that MIF null mice exhibit accelerated wound healing [28]. Grieb *et al*. observed that MIF levels are increased in the wound fluid of patients with acute and chronic wounds [29]. Higher MIF levels were found in exudates from acute wounds, which later healed uneventfully, than in exudates from chronic wounds.

In order to assess the functional role of MIF in acute wound healing disorders, we first focused on its ability to mobilize PBMCs to the site of inflammation. The ability of MIF to promote leukocyte recruitment was described earlier [30]. But in contrast to employing exogenously added cytokines, we used supernatants from whole adipose tissue that secrete a mixture of soluble factors containing stimulatory and inhibitory effects. MCP-1 is a well-known chemokine and served as a positive control [20]. We showed that PBMC chemotaxis was partially abrogated by MIF antibodies. Furthermore, our results indicate that MIF and MCP-1 may have an additive effect on PBMC chemotaxis. The chemotactic effect was not completely neutralized by the combined administration of anti-MIF and anti-MCP-1 antibodies implying that other soluble factors such as fatty acids may mediate the chemotaxis of inflammatory cells into adipose tissue [20, 31]. To underscore our *in vitro* observations, we investigated the accumulation of labeled macrophages into LPS-injected epididymal fat pads of WT and *Mif* ^*–/–*^mice. LPS was used as a stimulus to produce local adipose tissue inflammation [32, 33]. *Mif* ^*–/–*^mice showed a significantly lower infiltration rate of FITC-labeled macrophages when compared to WT mice which confirms the potential importance of MIF in cell recruitment to acutely inflamed adipose tissue. Our findings are in line with Verschuren *et al*. who observed decreased macrophage infiltration in white adipose tissue in *Mif*-deficient mice with lower expression of intercellular adhesion molecule (I-CAM)-1 and CD44, two relevant adhesion molecules inducing monocyte infiltration [34]. Although our immunohistochemical staining showed a higher ATM infiltration in IAT, an inflammatory-dependent increase of MIF expression in adipocytes or ATMs could not be demonstrated. Future studies are required to evaluate the source of MIF secretion in adipose tissue.

Finally, we studied the effect of adipose tissue-derived MIF on wound healing in an *in vitro* scratch assay. Results reveal a deleterious effect of IAT-derived MIF on fibroblast wound healing by influencing fibroblast migration. Based on the current literature, MIF shows a not fully understood discrepancy between the detrimental and beneficial effects on cutaneous wound healing and additional research is required to understand the specific actions of adipose-derived MIF in wound repair [35].

The present work represents an initial study to elucidate the role of MIF and the underlying mechanisms in cell recruitment during acute adipose tissue inflammation. Due to the small number of samples harvested from heterogeneous donor sites with varying backgrounds of the patients, the results must be interpreted with caution. The LPS used in our *in vivo* experiment must be regarded as a simplified model for acute adipose tissue inflammation ignoring the more complex wound healing disorders of the human samples. MIF antibodies did not completely inhibit PBMC chemotaxis, indicating that MCP-1, which had a stronger effect than MIF in our chemotaxis assay, and other chemokines are involved in this process. Our data, however, motivates to further investigate our observations in more extended studies. An *in vivo* wound model, preferably including a conditional knockout of *Mif*, may help to underscore the direct role of adipose-tissue-derived MIF in wound healing. Furthermore, more detailed investigations regarding MIF-ATM interactions, *i*.*e*. the role of MIF in ATM polarization, are desirable.

In conclusion, we showed that MIF mRNA and protein levels are increased in adipose tissue from inflammatory wounds. Our results additionally show that MIF appears to play a role in the recruitment of inflammatory cells to the side of acute adipose tissue inflammation and that MIF derived from inflamed adipose tissue may inhibit wound healing. Therefore, MIF may function as a possible target for future therapeutic approaches in impaired wound healing. However, further studies are required to shed light onto the explicit role of adipose-derived MIF during wound healing and the effect on ATM function.

## Supporting Information

S1 Data Raw Data of Experiments. (XLSX)
